# Sandwich-like electro-conductive polyurethane-based gelatin/soybean oil nanofibrous scaffolds with a targeted release of simvastatin for cardiac tissue engineering

**DOI:** 10.1186/s13036-023-00364-6

**Published:** 2023-07-06

**Authors:** Solmaz Saghebasl, Abbas Nobakht, Hesam Saghebasl, Sanya Hayati, Ozra Naturi, Reza Rahbarghazi

**Affiliations:** 1 Department of Medical Nanotechnology, Faculty of Advanced Medical Sciences, Tabriz University of Medical Sciences, Tabriz, Iran; 2 Research Center of Biosciences & Biotechnology (RCBB), University of Tabriz, Tabriz, Iran; 3 Faculty of Medicine, Islamic Azad University, Tabriz Branch, Tabriz, Iran; 4 Infectious and Tropical Diseases Research Center, Tabriz University of Medical Sciences, Tabriz, Iran; 5 Department of Organic and Biochemistry, Faculty of Chemistry, University of Tabriz, Tabriz, Iran; 6 Stem Cell Research Center, Tabriz University of Medical Sciences, Imam Reza St., Golgasht St, Tabriz, Iran; 7 Department of Applied Cell Sciences, Faculty of Advanced Medical Sciences, Tabriz University of Medical Sciences, Tabriz, Iran

**Keywords:** Electrospun scaffold, Electrospinning, Cardiac tissue Engineering, Polyurethane, Poly (glycerol sebacate), Soybean oil

## Abstract

Cardiac tissue engineering (CTE) is a promising way for the restoration of injured cardiac tissue in the healthcare system. The development of biodegradable scaffolds with appropriate chemical, electrical, mechanical, and biological properties is an unmet need for the success of CTE. Electrospinning is a versatile technique that has shown potential applications in CTE. Herein, four different types of multifunctional scaffolds, including synthetic-based poly (glycerol sebacate)-polyurethane (PGU), PGU-Soy scaffold, and a series of trilayer scaffolds containing two outer layers of PGU-Soy and a middle (inner) layer of gelatin (G) as a natural and biodegradable macromolecule without simvastatin (S) and with simvastatin (GS), an anti-inflammatory agent, were fabricated in the sandwich-like structure using electrospinning technique. This approach offers a combination of the advantages of both synthetic and natural polymers to enhance the bioactivity and the cell-to-cell and cell-to-matrix intercommunication. An in vitro drug release analysis was performed after the incorporation of soybean oil (Soy) and G. Soy is used as a semiconducting material was introduced to improve the electrical conductivity of nanofibrous scaffolds. The physicochemical properties, contact angle, and biodegradability of the electrospun scaffolds were also assessed. Moreover, the blood compatibility of nanofibrous scaffolds was studied through activated partial thromboplastin time (APTT), prothrombin time (PT), and hemolytic assay. The results showed that all scaffolds exhibited defect-free morphologies with mean fiber diameters in the range of 361 ± 109 to 417 ± 167 nm. A delay in blood clotting was observed, demonstrating the anticoagulant nature of nanofibrous scaffolds. Furthermore, rat cardiomyoblast cell lines (H9C2) were cultured on scaffolds for 7 days, and the morphology and cell arrangement were monitored. Data indicated an appropriate cytocompatibility. Of note, in the PGU-Soy/GS nanofibrous scaffold, a high survival rate was indicated compared to other groups. Our findings exhibited that the simvastatin-loaded polymeric system had positive effects on cardiomyoblasts attachment and growth and could be utilized as a drug release carrier in the field of CTE.

**Graphical Abstract:**

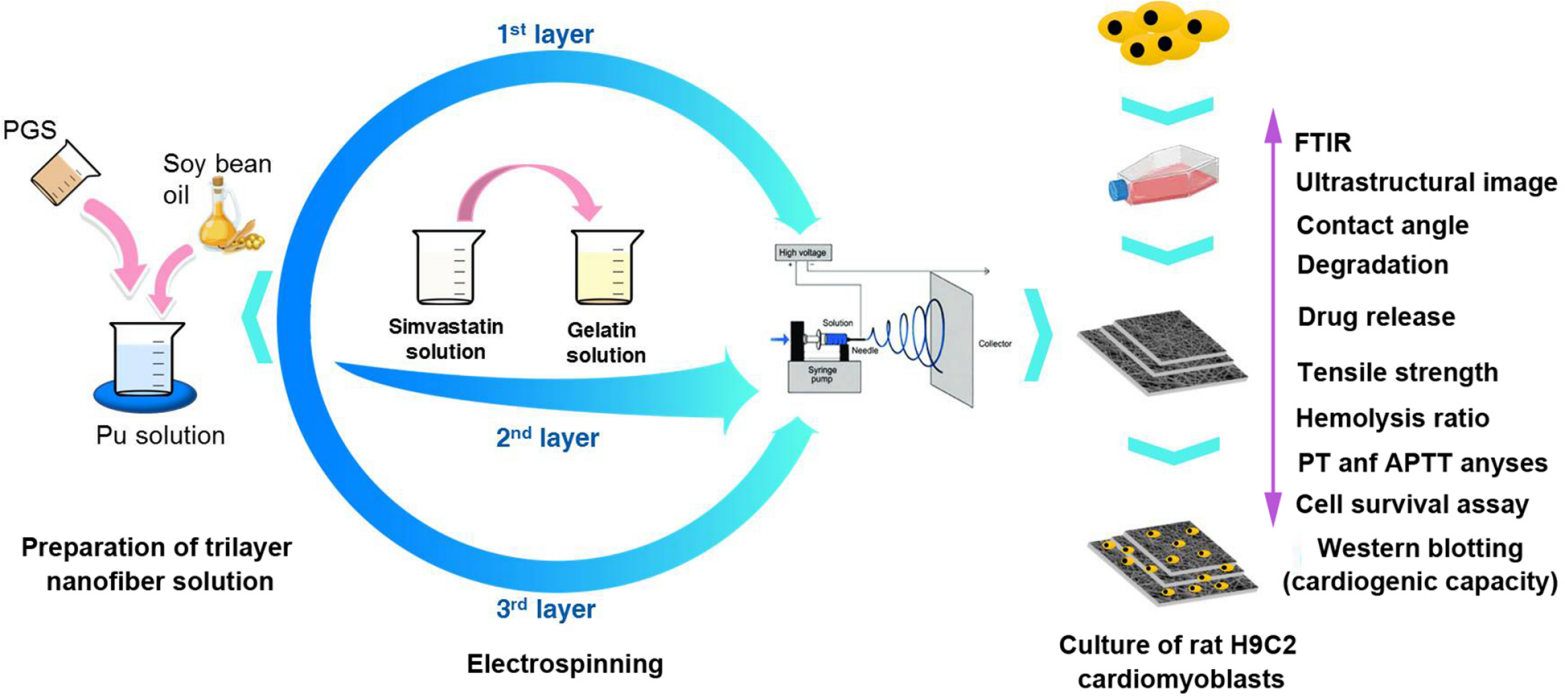

## Introduction

Cardiovascular diseases especially myocardial infarction are the major causes of human death in the world and currently are one of the most significant health challenges despite much progress in medicine [1, 2]. Recently, in vitro CTE offered new insights for the treatment of myocardial infarction, targeting the substitution of diseased tissue with a natural one grown into a 3D structure [3]. Despite the extensive use of electroconductive biomaterials to improve the physiological relevance of CTE, there are still some limitations related to bioactive engineering scaffolds with suitable mechanical properties and electrical conductivity for the regeneration of cardiomyocytes [3]. Therefore, the development of electroconductive biomaterials for the construction of scaffolds will be beneficial for CTE [4, 5].

Among various fabrication methods, electrospinning is an efficient approach to developing nanofibrous scaffolds mimicking the architecture of native tissue [6]. electrospinning could create highly porous and biomimetic niche for cell attachment, enabling the effective exchange of gases, nutrients, and wastes at the biological implant site [7]. Recently, considerable efforts have been concentrated on expanding the idea of using poly (glycerol sebacate) (PGS), a biodegradable and biocompatible polymer with excellent physical properties similar to an elastomer [8–10]. Despite the unique properties of PGS, the electrospinnability of neat PGS is limited due to its low molecular weight [11]. It has been proven that blending PGS with other synthetic or natural macromolecules can increase the viscosity of the polymer solution for the electrospinning procedure [12]. In the present study, polyurethane was used as a secondary polymer to increase the electrospinnability of PGS owing to their unique properties such as biocompatibility, biodegradability, processability, and elastic mechanical properties [13]. Modification of scaffolds with extracellular matrix (ECM) proteins such as gelatin can alter the surface properties of scaffolds and enhance their biocompatibility and biodegradability [14, 15].

Recent years have witnessed an upward trend in the use of polymeric drug delivery systems for tissue defect repair applications [16]. A significant application of drug delivery in tissue engineering (TE) is concerned with regulating the immune and inflammatory response to grafted scaffolds [17]. Statins are a group of cholesterol-lowering medications mainly used to reduce cardiovascular disease by preventing the synthesis of lipids in the liver [18, 19]. Statin-loaded scaffolds with proper release kinetic have been shown that can support tissue construction and control tissue-unfavorable reactions at the biological implant sites lowering inflammation-mediating cells [20].

Although conducting polymers have broad applications in biomedical engineering, they still display several technical limitations such as cytotoxicity, absence of cell attachment sites, and poor biodegradability and processability. Therefore, there remains an unmet demand to obtain typical biomaterials with biodegradable, biocompatible, and electroconductive properties. To address these limitations, a novel category of natural biodegradable biomaterial based on vegetable oils has been introduced to the scientific community that exhibits ideal electrical conductivity and inherent bioactivity on tissue cells in vitro and inflammatory response [21]. The electrical properties of bio-oils depend mainly on their molecular and chemical composition. The electrical conductivity of bio-oils is because of the presence of free charges which are under the influence of an electric field. These charges move to create an electric current [22]. Previous studies have shown that vegetable oils can promote biocompatibility and cell viability in other polymers [23, 24]. Among vegetable oils, Soy has gained great attention due to several desirable possessions such as superior biodegradability, excellent biocompatibility, non-toxicity, availability, and low price [25].

The main objective of the current work was to develop a trilayer (sandwich-like) electrospun scaffold based on PGU-Soy/GS as a potential drug delivery system to support rat H9C2 cardiomyoblasts with reconstructive capacity in vitro. To this end, a mixture of synthetic elastomers, PGU, has been chosen as the outer layer because of the dynamic characterizations and non-toxicity. Besides, gelatin was used as the middle layer to provide a platform for cell adhesion and proliferation. Finally, the morphology of drug-loaded electrospun scaffolds, mechanical properties, wettability, degradation tests, and biocompatibility of scaffolds was investigated.

## Materials and methods

### Materials

Glycerol, sebacic acid, sodium azide, acetic acid, dimethyl sulfoxide (DMSO), and simvastatin were obtained from Merck. For cell culture, phosphate-buffered saline (PBS), antibiotics including penicillin-streptomycin, Dulbecco’s modified eagle medium (DMEM), and fetal bovine serum (FBS), were supplied from GIBCO. Poly 4, 4’-methylene bis (phenylisocyanate)-alt-14-butanediol/dipropyleneglycol/polycaprolactone (Pu), gelatin powder (type A), Glutaraldehyde, 4′,6-diamidino-2-phenylindole (DAPI), 3,(4,5-dimethyithiazol-2-yl)2,5-diphenyl-tetrazolium bromide (MTT), tetrahydrofuran (THF), N, N-dimethylformamide (DMF), were purchased from Sigma-Aldrich. Soybean oil was produced by Eris Trading Company (Iran). The reagents used in APTT and PT assay and calcium chloride (CaCl_2_) solution were obtained from Fisher Scientific Company (USA). Live/Dead kit was obtained by Invitrogen. H9C2 cardiomyoblasts were obtained from Pasteur Institute (Iran).

### Fabrication of the electroconductive nanofibrous scaffolds

#### Synthesis of PGS

PGS prepolymer was obtained from condensation polymerization of sebacic acid and glycerol according to previous protocols [26]. Briefly, an equimolar ratio of sebacic acid and glycerol was heated at 120**˚**C under nitrogen for 24 h with rigid stirring. In the second step, the N_2_ bubbler was detached, and the reaction mixture was maintained in a vacuum oven for 48 h at 120**˚**C and 30 mTorr to eliminate excess water. The final product was a highly viscous polymer.

#### Solutions preparation

To prepare nanofibrous scaffolds, PGS/Pu solution with a concentration of 12% (w/v) with 1:2(w/w) ratios was dissolved in DMF and THF with the 4:1 (v/v) ratio and stirred overnight at RT. Then, a homogeneous solution was mixed with bio-oil (5% v/v) for at least 1 h before electrospinning. Gelatin solution (15% w/v) was prepared in acetic acid at RT. To prepare a gelatin-simvastatin (GS) solution containing 3 mg/ml simvastatin, the drug was added to double-distilled water and sonicated for 10 min [27, 28]. The solution was stirred overnight at RT. Then, a homogeneous GS electrospinning solution was prepared by blending the two solutions with stirring before electrospinning.

#### Fabrication of electrospun PGU-Soy/GS scaffold in a sandwich-like structure

The electroconductive trilayer scaffolds of PGU-Soy (inner and outer layers) and GS (middle layer) were produced using a simple electrospinning technique. To provide an optimum scaffold for cardiac regeneration, we optimized several electrospinning factors such as the applied electric potential, flow rate, distance between the needle and collector, inner needle diameter, as well as many solution parameters including polymers concentration, solvent, solution conductivity, and viscosity. In the first step, the PGU-Soy solution was delivered in 5 ml syringes. The electrospinning condition was fixed as follows: 18 KV voltage, 0.5 ml/h injection rate, and 15 cm distance. Then, the electrospinning parameters were adjusted at 20 KV, 0.6 ml/h flow rate, and 11 cm distance between needle and collector to electrospun gelatin solution (either with (GS) or without simvastatin) as the middle layer. The procedure was continued by the electrospinning of PGU-Soy solution on the gelatin layer using a similar protocol as described above.

### Characterization of nanofibrous scaffolds

#### SEM characterization

The morphology properties of the gold-sputter nanofibrous scaffolds before and after cell culture were investigated by FE-SEM (LEO 1430 VP, Germany). The fiber diameters were measured from FE-SEM micrographs by Image J software.

#### Functional group characterization

The FT-IR spectra of PGS, Pu, Soy, and the chemical characteristics of nanofibrous scaffolds were obtained by using FT-IR (Shimadzu 8400 S, Kyoto, Japan) in the range of 4000 − 400 cm^− 1^ and resolution of 4 cm^− 1^.

#### Wettability of the electrospun scaffolds

The hydrophilicity of electrospun nanofibrous scaffolds was measured by a contact angle measurement (PGX, SWEDEN). A droplet of deionized water was automatically dropped onto the PGU-Soy surface (outer layer) to determine the hydrophilicity of each sample.

#### Conductivity analysis

The electrical conductivity of nanofibrous scaffolds was evaluated using the four-point probe technique at ambient temperature.

#### Mechanical tests

The mechanical properties of the trilayer nanofibrous scaffolds were investigated with a tensile tester (ZO10, Zwick/Roell. Ulm, Germany). The fibers were cut in strips with a size of 50 × 10 mm^2^. The Tests were conducted with a 10 N load cell and a crosshead speed of 10 mm/min at RT. The tensile strength, Young’s modulus, ultimate tensile strength, and elongation at break were calculated from the stress-strain curve (Table 1).

**Table 1:** Physical properties of the electrospun nanofibers

Sample	Average fiber diameter (nm)	Conductivity (µs/cm)	Contact angel (˚)	Young’s modulus (Mp)	Ultimate tensile strength (Mp)	Elongation at break (%)
PGU	403 ± 119	9.55 × 10^− 5^	83 ± 3.40	10.00 ± 0.36	0.33 ± 0.04	3.45 ± 0.43
PGU-Soy	417 ± 167	0.54	61 ± 1.00	21.77 ± 0.20	0.72 ± 0.06	14.52 ± 0.47
PGU-Soy/G	361 ± 109	0.75	51 ± 1.37	12.25 ± 0.25	0.42 ± 0.02	15.00 ± 0.32
PGUSoy/GS	362 ± 83	0.80	33 ± 0.43	5.69 ± 0.28	0.43 ± 0.03	28.25 ± 0.26

#### In vitro degradation studies

The degradation rate of nanofibers was measured by incubating the nanofibrous scaffolds in phosphate-buffered saline (PBS) containing sodium azide 0.1% (w/v) at 37**˚**C under constant stirring for 28 days. At predetermined intervals, the nanofibers were washed in ultra-pure water, vacuum-dried at RT for a day, and then weighed for calculation of degradation rate according to the following equation:

$$Weight\;loss\;(\%)\;=\;wi-wt/wi\times100$$

*wi* and *wt* are the initial and degraded weights of nanofibrous scaffolds, respectively.

#### In vitro drug release tests

The release of simvastatin (S) from the electrospun PGU-Soy/GS scaffold was done by incubating a known fiber mass in 25 ml of PBS (pH = 7.4) at 37**˚**C under constant stirring for 9 days. At predetermined time intervals, 3 ml of buffer solution was withdrawn to measure drug release using a Shimadzu 2550 UV spectrophotometer at 231 nm.

#### Blood compatibility assessment of nanofibrous scaffolds

In the present study, the hemolysis assay was conducted to measure the effects of nanofibrous scaffolds on red blood cell lysis. Firstly, electrospun samples (~ 1 cm^2^ area) were placed in microtubes and 800 µl of normal saline solution (0.9% w/v) and 200 µl of citrated blood were overlaid [29]. In the control groups, 200 µl diluted blood with 800 µl distilled water was used (positive control). In this study, 1 ml of normal saline solution was used as a negative control. Afterward, all tubes were placed in an incubator for 1 hour at 37**˚**C and then centrifuged at 3000 rpm for 10 minutes. Finally, the absorbance of supernatants was read by a microplate reader at 545 nm and the hemolysis rate was obtained as follows:

$$Hemolysis\%=ODSample-ODNegativecontrol/ODPositivecontrol-ODNegativecontrol\times100$$

Moreover, APTT and PT assays were carried out to measure the anticoagulant behavior of the nanofibrous scaffolds. Electrospun mats with a dimension of 1 × 1 cm^2^ were incubated in PBS for 30 min at 37˚C before starting the APTT assay. Then, 50 µl platelet-poor plasma was placed on the nanofibrous samples and incubated at 37˚C for 1 min. After that, 50 µl APTT reagents were added and incubated at 37˚C for the next 3 min. The procedure was followed by adding 50 µl CaCl_2_. Similarly, 50 µl platelet-poor plasma was mixed with 50 µl thromboplastin reagent for the PT assay, and the coagulation time was measured quickly using a chronometer.

### Cell culture study

Before culturing rat H9C2 cardiomyoblasts on the nanofibrous scaffolds, fiber masses were disinfected by soaking in 70% ethanol solution for 30 min, followed by exposing scaffolds to UV radiation for 2 h. Thereafter, nanofibrous scaffolds were rinsed there times with PBS solution and incubated in the culture media (DMEM, glucose (1 g/L), 10% FBS, 1% Penicillin-Streptomycin) under standard conditions (humidified atmosphere, at 37**˚**C, and 5% CO_2_). Finally, a total number of 1 × 10^5^ H9C2 cardiomyoblasts was seeded onto nanofibrous scaffolds and placed in a standard humidified incubator (Innova Co-170, USA) for 7 days. The culture medium was refreshed every 3–4 days.

#### Cell morphology

After 7 days, the morphology and attachment of H9C2 cardiomyoblasts cultured on nanofibrous scaffolds were investigated by FE-SEM. Briefly, after 7 days of cell culture, samples were fixed with 2.5% glutaraldehyde solution overnight and rinsed in PBS. Subsequently, cell dehydration was carried out in different EtOH concentrations (50, 70, 90, and 100%). Finally, the structure of fibers and morphology were monitored by FE-SEM.

#### Cell proliferation studies using MTT assay

The viability of cultured H9C2 cardiomyoblasts was assessed after 2, 4, and 6 days of cell seeding in 96-well plates using an MTT assay. At each time point, the supernatant was eliminated from each well, and MTT solution (5 mg/ml) was added to each sample. After 4 h of incubation at 37**˚**C, the supernatant was removed, and formazan crystals were dissolved in DMSO. The purple solution was then pipetted out into another 96-well culture plate, and the absorbance of each sample was determined by a spectrophotometric plate reader (MK3, Thermo Electron Corporation, USA) at a wavelength of 570 nm. The H9C2 cardiomyoblasts cultured on blank tissue culture plates (TCP) were employed as the control substrates. The cell viability rates were expressed as % of control. Moreover, a live/dead cell cytotoxicity assay was conducted to evaluate the viability of H9C2 cells seeded on nanofibrous scaffolds after 7 days. Calcein-AM was applied to stain live (green) cells. After the completion of the culture period, the cell-seeded nanofibrous scaffolds were fixed in 4% paraformaldehyde and sectioned using cryo-sectioning apparatus (Leica). Sections were stained with 1 µM Calcein-AM and 1 µg/ml 4, 6-diamidino-2-phenylindole (DAPI). The fluorescent images on the nanofibers were observed using fluorescence microscopy (Olympus BX51).

#### Western blotting

To monitor the maturation of plated H9C2 cells on electrospun fibers, protein levels of α-actinin, Connexin-43, and myosin were studied using western blotting. 7 days after in vitro culture, cells were collected in different groups and lysed using RIPA buffer. Protein samples were electrophoresed and separated using 10% SDS-PAGE electrophoresis. The procedure was continued by the transfer of samples to PVDF membranes and incubated with anti-myosin (Cat No: ab2480; Abcam), anti-Connexin-43 (Cat no: sc-13,558; San Cruz Biotech. Inc.), anti-α-actinin (Cat no: sc-130,928; San Cruz Biotech. Inc.) for 1 h at RT. After several washes with PBST, membranes were incubated with HRP-tagged secondary antibodies (Cat no: sc-2357; Cat no: 516,106; San Cruz Biotech. Inc.) for 1 h at RT. To visualize the bands, X-ray films, and ECL solution was used. β-actin was considered a housekeeping protein (Cat no: sc-47,778; San Cruz Biotech. Inc.).

### Statistical analysis

One-Way ANOVA with Tukey post-hoc analysis was performed for statistical comparisons between different groups and p < 0.05 was taken as significant. The experimental results were expressed as mean ± standard deviations (n = 3).

## Results and discussion

### SEM studies

**Fig. 1:**
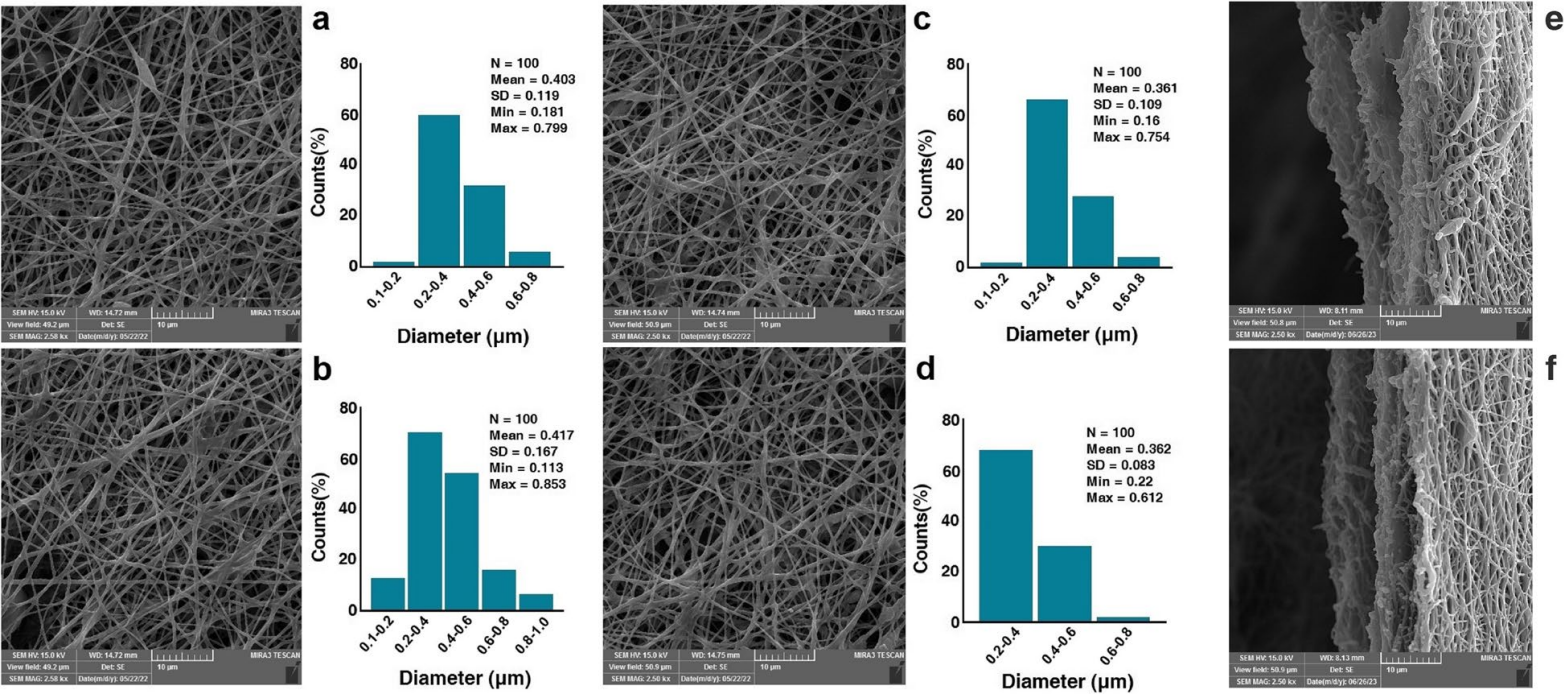
The morphological properties of electrospun (**a**) PGU, (**b**) PGU-Soy, (**c**) PGU-Soy/G, (**d**) PGU-Soy/GS nanofibers (scale bars: 10 μm) and the corresponding diameter distributions, the cross-section of (**e**) PGU-Soy/G, (**f**) PGU-Soy/GS nanofibers (scale bars: 10 μm). The inner and outer layers are PGU-Soy while the middle layer is composed of simvastatin-loaded or –free gelatin

The microstructural properties of scaffolds have a remarkable effect on the distribution of cells, nutrient diffusion, and cellular metabolism [30]. A highly porous interconnected architecture is essential for tissue reconstruction. Highly porous architectures provide suitable interaction between cells and scaffolds [31]. Therefore, studying the morphology of electrospun scaffolds to confirm a uniform structure is a crucial issue in TE research. Figure 1 represents the FE-SEM micrographs of the electrospun fibers and the corresponding diameter distribution. The SEM image of the PGU nanofibrous scaffold exhibited randomly-oriented fibers (Fig. 1(a)). Data indicated that the electrospun nanofibers were well-defined, without any defects. Based on the data, the mean diameter of PGU fibers reached 417 ± 167 nm. The incorporation of Soy oil into this mixture resulted in the reduction of fiber diameter. As shown in Fig. 1(b), the mean diameter of the electrospun PGU-Soy fibers was 403 ± 119 nm, which is less compared to PGU fibers. Our data were in support of previously conducted experiments associated with electrospun polyurethane scaffolds containing castor oil [32]. One reason would be related to the increased conductivity of the solution mixture during the integration of Soy oil into the polymer matrix. It was noted that the smaller fiber diameter can be favorable for cardiomyocyte adhesion and proliferation due to their greater specific surface area [33]. We found that non-significant differences in the mean diameters of PGU and PGU-Soy nanofibers (Fig. 1a-b). Figure 1 (panels c and d) indicates the SEM images of sandwich-like PGU-Soy/G fibers before and after adding simvastatin. It was observed that sandwich-like nanofibrous scaffolds exhibited reduced fiber diameter compared to the PGU-Soy scaffold. The mean fiber diameter for PGU-Soy/GS scaffolds before and after incorporation with simvastatin was 361 ± 109 and 362 ± 83 nm, respectively. Our results indicated that the incorporation of simvastatin did not exhibit a significant influence on the size of the diameters. Furthermore, cross-sectional images of PGU-Soy/G and PGU-Soy/GS nanofibrous scaffolds were presented in Fig. 1(e, f). As can be observed from cross-sectional views, the arrangement of multilayers was uniform and tight, which exhibited a desirable sandwich-like structure.

### FT-IR analysis

**Fig. 2:**
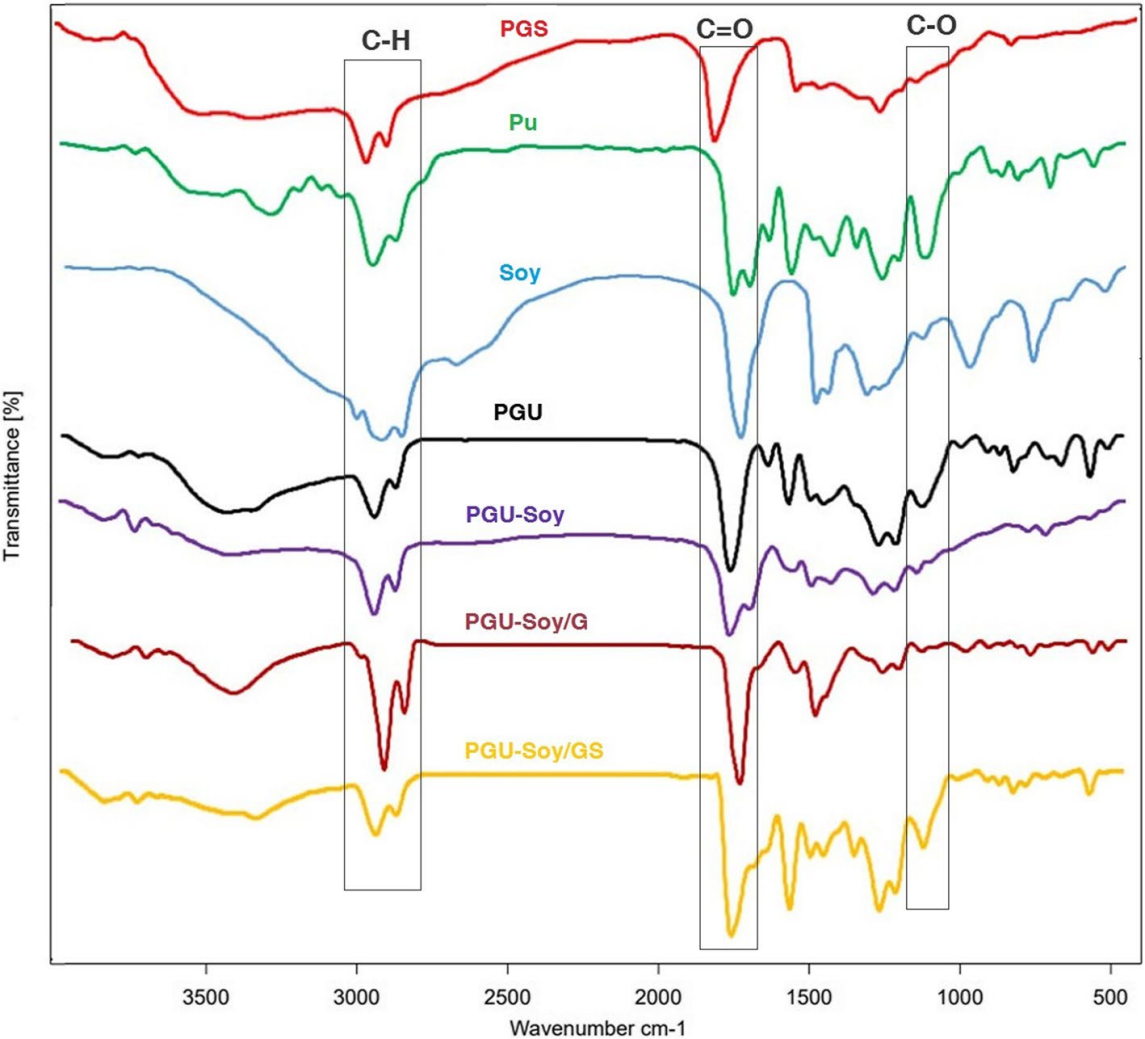
FT-IR spectra of pure PGS, Pu, Soy, and electrospun PGU, PGU-Soy, PGU-Soy/G, and simvastatin loaded PGU-Soy/G nanofibers

The Chemical structure of synthesized PGS, as well as the nanofibrous scaffold, was investigated using FT-IR analysis. In the PGS spectrum (Fig. 2), the strong absorption peak at 1735 cm^− 1^ exhibits the presence of ester carbonyl groups (C = O), and the peaks at 2925, and 2856 cm^− 1^ are related to CH_2_ stretching. The broad absorption band around 3474 cm^− 1^ is attributed to OH stretch vibration. The peak at 1373 cm^− 1^ is indicative of the C-O-C stretching. There is a band at 1169 cm^− 1^, representing carbonyl stretching vibration (C-O). The peak at 3300 cm^− 1^ in the pure Pu spectrum indicates the NH stretching, and the peak at 1532 cm^− 1^ is representative of the vibration of CHN. The peak at 1223 cm^− 1^ shows coupled C-N and C-O stretching, and the twin peak at 1735 and 1671 cm^− 1^ is due to the presence of C = O stretching in the Pu structure. The peaks at 2865 and 2942 cm^− 1^ can be assigned to the symmetric and asymmetric CH_2_ stretching, and the peak at 1086 cm^− 1^ is related to C-O stretching. FT-IR spectra of pure soybean oil, as well as PGU, PGU-Soy, PGU-Soy/G, and PGU-Soy/GS nanofibrous scaffolds, are also shown in Fig. 2. As can be seen, carbonyl groups are present in the structure of all electrospun samples and the chemical composition of all electrospun scaffolds consist of the same functional groups.

### Wettability of the electrospun scaffolds

**Fig. 3:**
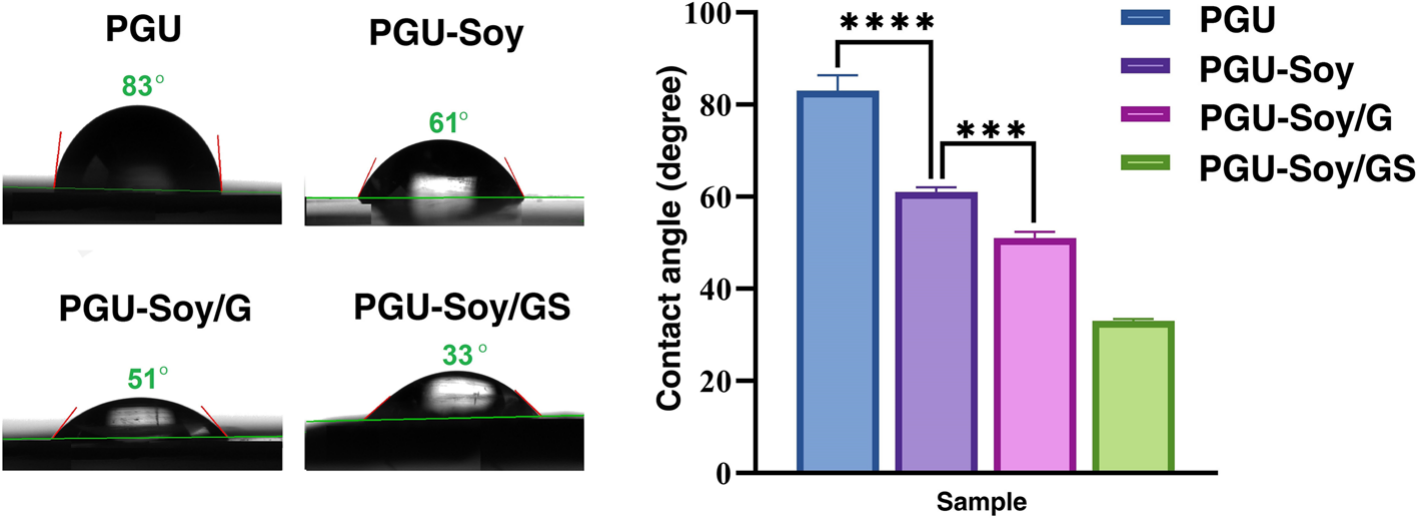
Water contact angle images and graph for electrospun nanofibers. One-Way ANOVA with Tukey post hoc analysis. ***p < 0. 001 and ****p < 0.0001

The wettability of scaffolds is one of the most important indexes in TE applications, mainly due to its influences on protein adsorption, cell attachment, and proliferation. Figure 3 shows the images of the water contact angles on prepared nanofibrous scaffolds. As can be seen, the contact angle (CA) of the PGU nanofibrous scaffold was found to be 83 ± 3.4˚, whereas the PGU-Soy scaffold was around 61 ± 1˚, which showed a greater hydrophilic behavior. The incorporation of Soy oil resulted in a lower contact angle, indicating an increase in wettability with a decrease in the fiber diameter of the PGU-Soy scaffold. Similar results were reported by previous works that incorporating bio-oil into polyurethane scaffolds leads to an increase in wettability [34]. The water contact angle images of trilayer PGU-Soy/G and PGU-Soy/GS nanofibrous scaffolds were shown in Fig. 3. The results showed that the water contact angle for PGU-Soy/G and PGU-Soy/GS scaffolds was around 51 ± 1.37º and 33 ± 0.43º, respectively. The enhanced wettability of sandwich-like scaffolds was due to the presence of the carboxyl and amine functional groups in the structure of gelatin. Besides, the functional groups of hydroxyl and carbonyl in the structure of simvastatin have increased the wettability of PGU-Soy/GS fibers. This provides a more appropriate environment for cellular responses and cell-scaffold interactions, which has been reported previously.

### The electrical conductivity analysis

The electrical conductivity of fabricated scaffolds is urgent for the stimulation of cardiac muscle cells. The results show that PGU fibers do not exhibit detectable electrical conductivity (Table 1). The incorporation of Soy oil with nanofibrous scaffolds resulted in a remarkable improvement in electrical conductivity. In comparison to PGU fibers, PGU-Soy, PGU-Soy/G, and PGU-Soy/GS nanofibrous scaffolds revealed conductivity of 0.54 µs/cm, 0.75 µs/cm, and 0.80 µs/cm respectively.

### Mechanical tests

**Fig. 4:**
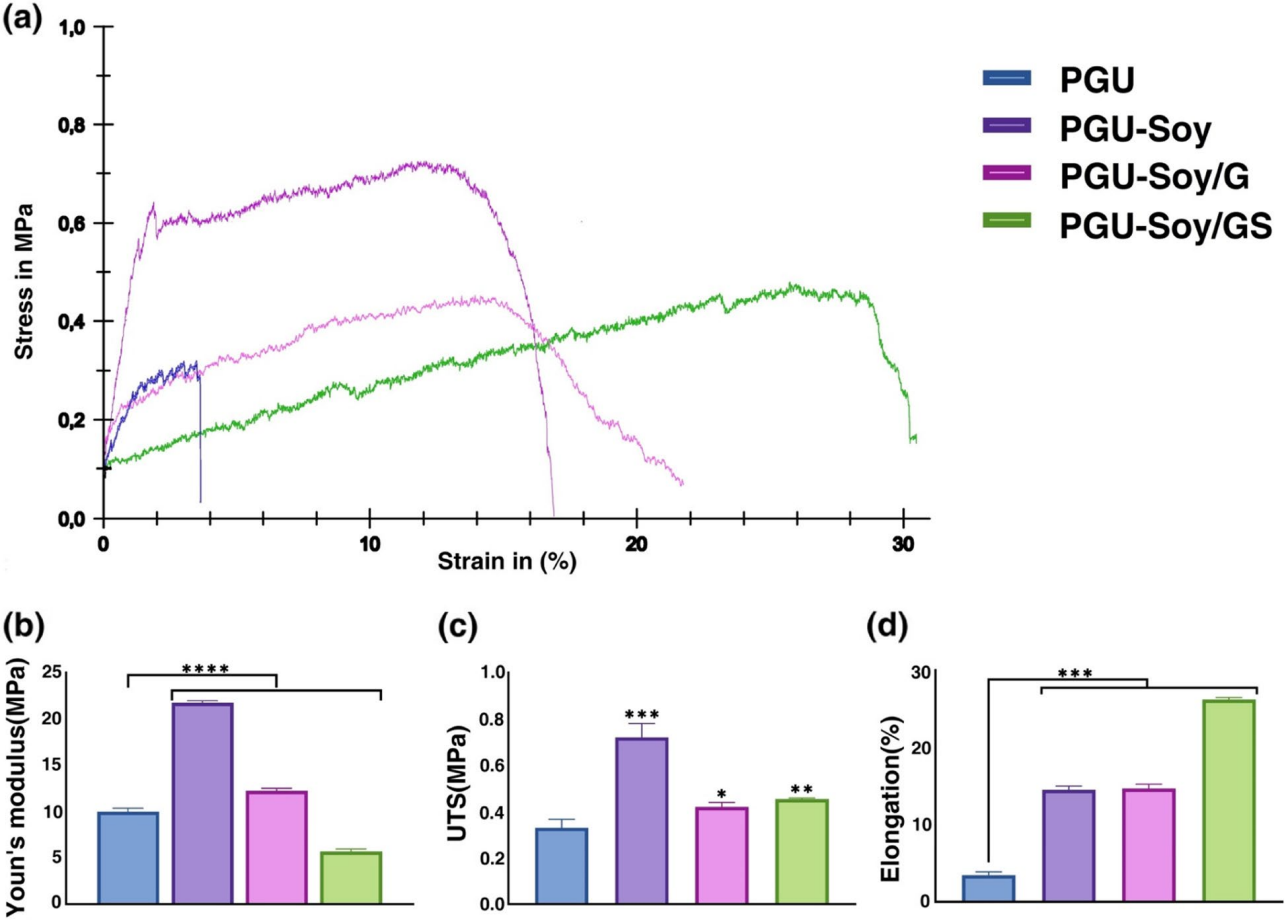
Mechanical characterization of the prepared scaffolds. **a** Stress-strain curves of electrospun nanofibers, (**b**) Young’s modulus, (**c**) ultimate tensile strength, and (**d**) elongation at break of electrospun nanofibers. One-Way ANOVA with Tukey post hoc analysis. *n* = 3; *p < 0.05, **p < 0.01, ***p < 0.001, and ****p < 0.0001

The mechanical features of electrospun scaffolds are another critical parameter in TE due to their effects on various aspects of cell culture like cell attachment, spreading, and signaling. Results of tensile testing of fabricated nanofibrous scaffolds are presented in Table 1, and Fig. 4, respectively. According to stress-strain outcomes, the tensile strength of the PGU nanofibrous scaffold blended with Soy oil was greater than that of the PGU scaffold. The electrospun PGU scaffold exhibited an ultimate tensile strength of 0.33 ± 0.04 MPa, Young’s modulus of 10 MPa, and a failure strain of 3.45 ± 0.43%. As can be seen, the incorporation of Soy oil with the nanofibrous scaffold resulted in the increase of Young’s modulus, ultimate tensile strength, and also failure strain to 21.77 ± 0.2 MP, 0.72 ± 0.06 MPa, and 14.52 ± 0.47%, respectively. It is suggested that the increase of strain strength can be attributed to the adhesive properties of Soy oil. Similar outcomes were reported by Jaganathan and co-workers, in which the introduction of castor oil or turmeric oil into an electrospun Pu nanofibrous scaffold resulted in an enhancement in the tensile strength [32, 34]. Moreover, Unnithanet al. reported similar results for electrospun Emu oil/Pu scaffolds for the regeneration of wound skin tissue [35]. The addition of gelatin to nanofibrous scaffolds caused a reduction in Young’s modulus and ultimate tensile strength. This reduced mechanical strength can be attributed to the poor physical properties of gelatin. Furthermore, when simvastatin was blended with gelatin, Young’s modulus of the electrospun scaffold significantly was reduced from 12.25 ± 0.25 MP to 5.69 ± 0.28 MP. In contrast, the failure strain increased from 15 ± 0.32 to 28.25 ± 0.26, indicating good flexibility and deformability. The tensile test data reported in this study was in agreement with the previous works in CTE [36, 37]. It is worth mentioning that the mechanical properties of all electrospun scaffolds are significantly greater than those of the native human myocardium, indicating a tensile strength in the range of 3KPa, and Young’s modulus between 0.02 and 0.5 MPa [38]. It should be noted that one of the purposes of preparing fiber structures is to use them as cardiac patches. Therefore higher values of Young’s modulus and tensile strength not only do not harm cardiac patches but can also reduce infarction.

### In vitro degradation analysis

**Fig. 5:**
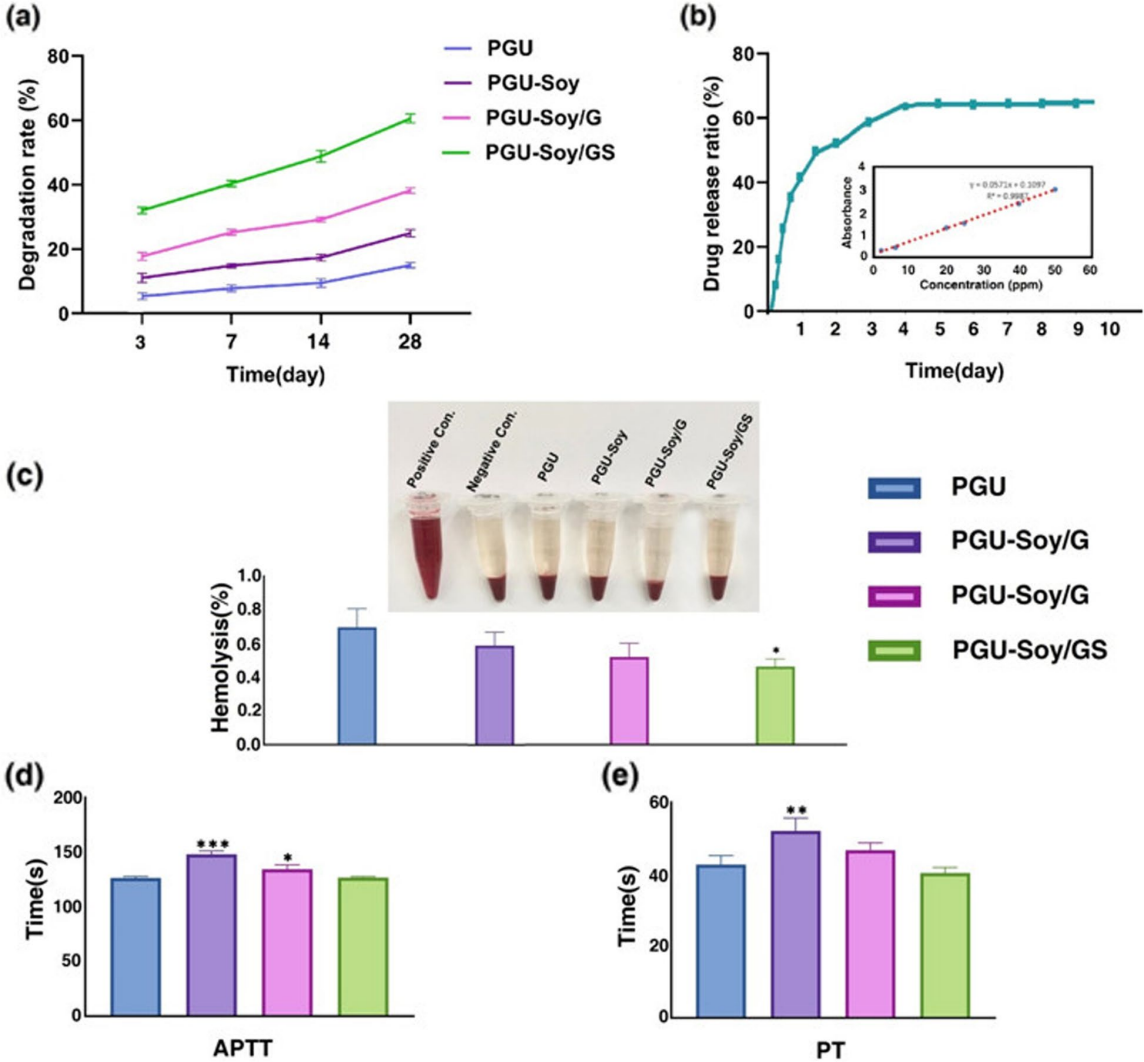
**a** In vitro degradation of electrospun nanofibers in 5 ml of PBS (PH = 7.4) containing 0.1% sodium azide at 37ºC under constant stirring for 28 days. **b** Simvastatin release profiles of electrospun nanofibers in PBS (PH = 7.4) at 37ºC during 9 days and a calibration curve of simvastatin in PBS solution. **c** The hemolysis ratio of electrospun scaffolds, blood in normal saline (NaCl 9%) was chosen as the negative control, and blood solution in deionized water was chosen as the positive control. **d** Activated partial thromboplastin time (APTT) of electrospun scaffolds. **e** Prothrombin time (PT) of electrospun scaffolds. One-Way ANOVA with Tukey post hoc analysis. n = 3. *p < 0.05, **p < 0.01, and ***p < 0.001

The degradation studies were performed to determine the corrosion rate of the fibers in the cell culture condition. The results revealed that all electrospun nanofibers indicated progressive weight loss during 28 days. According to Fig. 5(a), the incorporation of gelatin and simvastatin showed significant impacts on the degradation rate of PGU-Soy/GS fiber. This phenomenon can be explained by more hydrophobicity of gelatin and simvastatin and consequently more effective collisions with water molecules.

### In vitro release behavior

Drug delivery systems have emerged as a new process to increase the efficiency of scaffolds used in TE and can help improve the therapeutic process. Therefore, drugs, genes, and growth factors can be loaded into the polymeric solution to develop a controlled drug delivery system [39]. The drug release profile of the electrospun PGU-Soy/GS scaffold during 9 days is presented in Fig. 5 (b). Based on the results, the scaffold loaded with simvastatin represented an initial burst release of about 45% within 1 day. It then reached a maximum release of 60% within 5 days with a slower rate of release and finally achieved a plateau, in which a drug was released at a constant rate. As can be observed, the electrospun nanofibrous scaffold loaded with simvastatin revealed a triphasic release profile. This burst release is common in drug-loaded nanofiber scaffolds and could be attributed to the solubility and quantity of drugs that release from the scaffold and was dependent on the transport properties of the system and geometry. For example, Rezk et al. indicated that about 20% simvastatin was released from PCL-PGS-HA nanofibers within 1 day and then reached a maximum release of 60% within 7 days. In our system, smaller diameter fibers resulted in higher initial burst release. Based on previous studies, the release of drugs in fibers with fine diameters is faster compared to coarse fibers. Consequently, large-diameter fibers seemed to be favorable for slow and long-term drug release [40].

### Blood compatibility assessment of nanofibrous scaffolds

The hemolysis rate is the best method to analyze the biocompatibility of nanofibrous scaffolds against red blood cells. In agreement with international standards, hemolytic values less than 2% are considered blood-biocompatible and non-hemolytic materials, while a value above 2% is classified as hemolytic compounds [41]. In Fig. 5(c), the hemolytic percentage of nanofibrous scaffolds is shown. In this study, the prepared nanofibers displayed a hemolytic index of less than 1%, indicating high blood compatibility. Moreover, the blood clotting and anticoagulant nature of nanofibrous scaffolds were calculated by APTT and PT assays. The APTT was utilized to investigate the intrinsic pathway and the PT was applied to investigate the extrinsic pathway. As can be seen in Fig. 5(d, e), in contrast to the PGU scaffold, APTT and PT values of PGU-Soy nanofiber represented prolonged blood clotting time. This increase in the blood clotting time of nanofiber may be attributed to the smaller diameter of PGU-Soy nanofiber and also its better surface properties, resulting in the enhancement in anticoagulant nature. As reported in previous studies [42], scaffolds with smaller fiber diameters could favor increased blood compatibility by delaying the blood clotting time. In our study, the coagulation time of the PGU-Soy/G scaffold was found to be reduced compared to PGU-Soy nanofiber owing to the incorporation of gelatin. This reduction in coagulation time may be due to hydrophilic properties and the contact angle of this scaffold [43, 44]. Moreover, the addition of simvastatin to the PGU-Soy/G nanofiber resulted in a slight decrease in coagulation time but it was similar range to the PGU-Soy scaffold.

### Cell-scaffold interactions

**Fig. 6:**
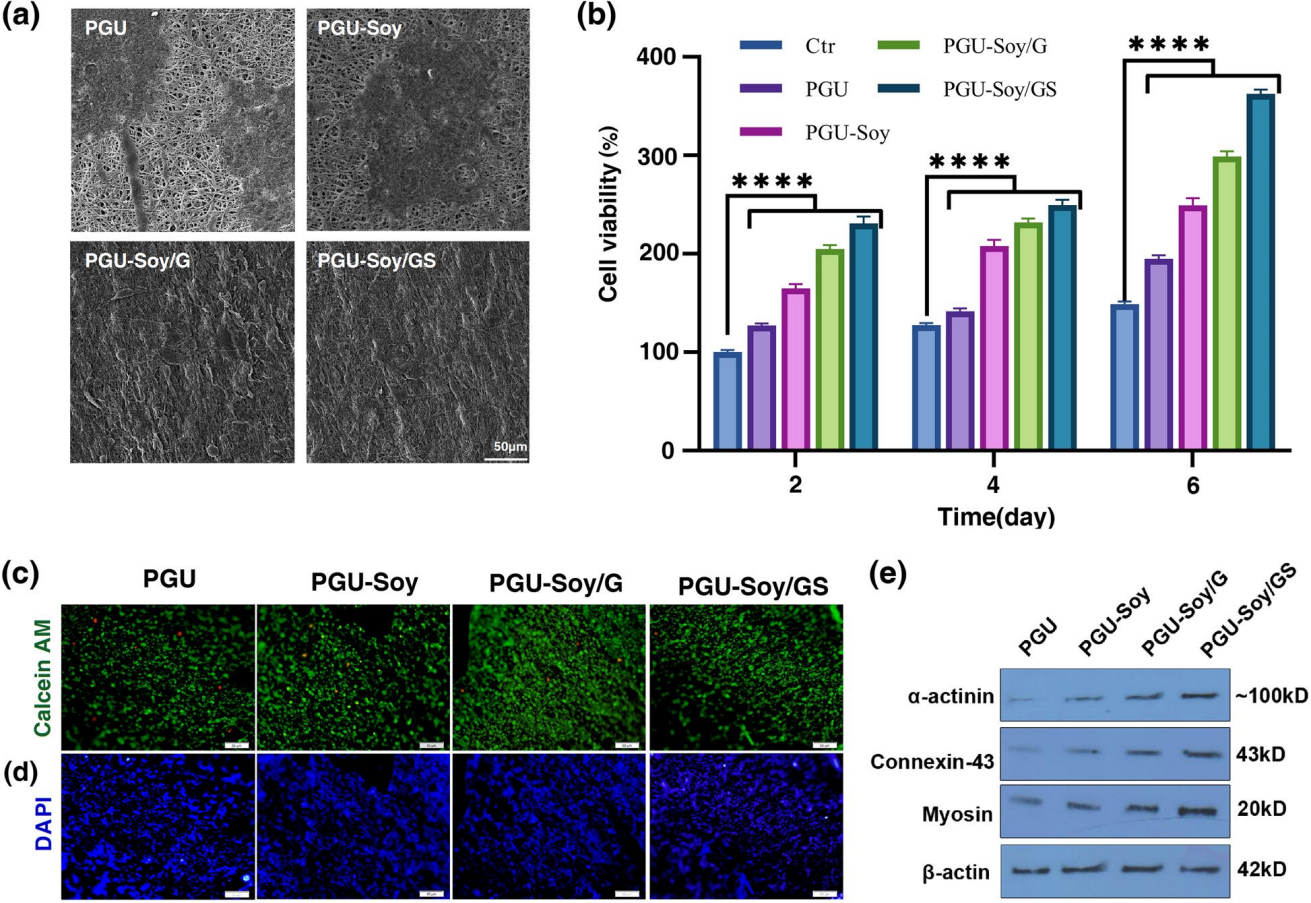
Cellular studies. **a** FE-SEM micrographs of seeded H9C2 cells on electrospun nanofibers after 7 days of culture (scale bar: 50 μm). **b** H9C2 viability on electrospun nanofibers (****p < 0.0001). **c** Live-Dead staining images of H9C2 cells at 7 days after treatment with electrospun nanofibers (live: green, dead cells: red, scale bar:50 μm). **d** DAPI staining of rat H9C2 cells after 7 days of cell culture on electrospun nanofibers (scale bar:50 μm). Measuring protein levels of (**e**) α-actinin, Connexin-43, and myosin protein levels of the H9C2 cells on the nanofibrous scaffolds using western blotting. One-Way ANOVA with Tukey post hoc analysis. ****p < 0.0001

The morphology of rat H9C2 cardiomyoblasts on the electrospun PGU, PGU-Soy scaffolds, and PGU-Soy/G scaffold in the presence and absence of simvastatin after 7 days are shown in Fig. 6 (a). In the present work, cell growth studies in the outer or inner layer were not compared. Instead, we studied the influence of simvastatin on cellular adhesion and survival. Weak cellular attachment on the Pu surface and high adhesion on the gelatin surface have been studied by other investigators [45]. As can be seen in Fig. 6 (a), after 7 days of seeding, the cells can attach and spread on the surface of all nanofibrous matrices, but the simvastatin-loaded sample indicated a greater spreading and proliferation during the same period. In this study, we observed that all the nanofibrous scaffolds promoted H9C2 cell adhesion and proliferation. The incorporation of gelatin as a natural polymer and simvastatin with PGU-Soy matrices provides good hydrophilicity, enhanced bioactivity, and high cell affinity with the consistent release of simvastatin from PGU-Soy/GS scaffold for tissue regeneration. This provides a more favorable environment for H9C2 cardiomyoblast attachment and proliferation. The viability of rat H9C2 cardiomyoblasts on nanofibrous scaffolds was assessed by MTT assay after 2, 4, and 6 days of cell seeding (Fig. 6(b)). The results revealed that cell viability was enhanced with time in cells plated on electrospun scaffolds. After 6 days of cell culture, survival rates of H9C2 cells on PGU-Soy/GS nanofibrous scaffolds were significantly increased compared to the PGU nanofibrous scaffold. Results clearly showed that gelatin and simvastatin highly accelerate H9C2 viability on PGU-Soy/GS nanofibrous scaffold. Based on the release panel, it seems that the release of simvastatin has a positive effect on the survival of plated H9C2 cells. As shown in Fig. 6(c), green fluorescent points indicating viable cells were evenly distributed on nanofibrous scaffolds. These results represented that H9C2 cells can attach and undergo dynamic growth, confirming the good cytocompatibility of the nanofibrous scaffolds. Finally, as shown in Fig. 6(d), the cell spreading behavior of H9C2 cardiomyoblasts on electrospun scaffolds was confirmed by DAPI staining results. Western blotting indicates that the plated cells can acquire specific phenotypes similar to mature cardiomyocytes. Based on the data, the protein levels of α-actinin, Connexin-43, and myosin were increased in PGU-Soy/GS scaffold and had superiority related to PGU, PGU-Soy, and PGU-Soy/G groups. We noted the minimum cardiogenic effects in the PGU scaffold compared to the other groups. The therapeutic effects of PGU-Soy, and PGU-Soy/G groups were more than the PGU scaffolds. These features indicate that the loading of simvastatin on the PGU-Soy/G scaffold can improve the cardiogenic outcomes.

## Conclusions

In this research, we designed four different types of electrospun scaffolds based on PGU, PGU-Soy, PGU-Soy/G, and simvastatin-loaded PGU-Soy/G scaffold as a potent in vitro. The morphology of electroconductive scaffolds, mechanical characterization, wettability, electroconductivity properties, degradation tests, and biocompatibility of scaffolds were evaluated. An In vitro drug release analysis was performed to investigate the sustained release potential of the simvastatin-loaded scaffold. Our results showed that blending Soy oil as a semiconducting material with nanofibrous scaffolds improved the electrical conductivity of nanofibrous scaffolds. In addition, SEM micrographs exhibited randomly oriented and defect-free nanofibers in the range of 361 ± 109 to 417 ± 167 nm. It was observed that the PGU-Soy nanofibers delayed blood clotting time more than the PGU, showing improved blood compatibility. Also, the nanofibrous scaffolds displayed a hemolytic index of less than 1%, indicating high blood compatibility. Moreover, cellular behavior studies revealed that incorporating gelatin with nanofibrous scaffolds promoted cell growth, spreading, wettability, and the biodegradation rate of fabricated scaffolds. Furthermore, simvastatin had a positive effect on cell adhesion, growth, and functional maturation. These results demonstrated a favorable interaction between electrospun scaffolds with the H9C2 cardiomyoblasts and recommend its potential application in TE as a cardiac substitute.

## Data Availability

All data generated or analyzed during this study are included in this published article.

## Abbreviations

APTT: Activated partial thromboplastin time
CTE: Cardiac tissue engineering
DMEM: Dulbecco's Modified Eagle Medium
FBS: Fetal bovine serum
G: Gelatin
MI: Myocardial Infarction
MWCNTs: Multi-Walled Carbon Nanotubes
PBS: Phosphate-buffered saline
PGS: Poly glycerol sebacate
PGU: Poly (glycerol sebacate)-polyurethane
PT: prothrombin time
Pu: Polyurethane
S: Simvastatin
Soy: Soybean oil
TE: Tissue Engineering
